# Functional Specialization of the Visual Word Form Area During Word Reading: A Multimodal Neuroimaging Study

**DOI:** 10.1162/NOL.a.225

**Published:** 2026-03-26

**Authors:** Lala Gu, Huiling Li, Jing Qu, Jingyu Yang, Xin Fu, Rui Hu, Leilei Mei

**Affiliations:** Philosophy and Social Science Laboratory of Reading and Development in Children and Adolescents, Ministry of Education, South China Normal University, Guangzhou, China; Center for Studies of Psychological Application, South China Normal University, Guangzhou, China; School of Psychology, South China Normal University, Guangzhou, China; School of Psychology, Guizhou Normal University, Guiyang, China; Key Laboratory of Behavioral and Mental Health of Gansu, Northwest Normal University, Lanzhou, China

**Keywords:** visual word form area, word reading, neural representation, connectivity-biased hypothesis, fMRI

## Abstract

The visual word form area (VWFA) has been consistently identified as a crucial structure in word reading, and its function differs across subregions. Nevertheless, the functional roles of its subregions and their functional origins remain controversial. Here, we adopted multimodal neuroimaging techniques (i.e., task-state fMRI, resting-state fMRI, and diffusion MRI) combined with representational similarity analysis to investigate the functional role of VWFA subregions and the brain circuitry supporting their function in two experiments. Results revealed respective roles of the posterior and anterior VWFA subregions in visual and semantic processing, which is consistent with their respective connectivity to orthographic and semantic networks. In addition, processing demands modulated the neural representations of high-level linguistic information in the VWFAs. These convergent findings elucidated the local neural computations in the VWFAs and their cooperative mechanism with distant brain regions related to language processing, jointly providing multimodal neuroimaging evidence for the connectivity-biased hypothesis.

## INTRODUCTION

The visual word form area (VWFA), which is located in the middle of left ventral occipitotemporal cortex (lvOT), has been consistently suggested to play a vital role in visual word reading (Cohen & Dehaene, 2004; Haugg et al., 2025; Price & Devlin, 2003; Xue et al., 2023). Specifically, previous studies have found that VWFA exhibited stronger activation for words compared to non-words or other visual materials, such as faces or objects (Cohen & Dehaene, 2004; Glezer & Riesenhuber, 2013). In addition, it has been revealed that neural activation in VWFA was associated with reading performance in both children and adults (Centanni et al., 2019; Kubota et al., 2019). In contrast, the interactive account of VWFA has proposed that this area integrates low-level visual information and high-level lexical information (Price & Devlin, 2011) and consequently represents the information of orthography, phonology, and semantics (Gu et al., 2024; Liu et al., 2023). Further research found that the VWFA exhibited shared activation during the word reading and the naming of their corresponding pictures, suggesting that VWFA is not exclusively specialized for visual word processing (Neudorf et al., 2022). Further research has demonstrated that this area is a highly heterogeneous structure, and its posterior and anterior subregions have dissociable functions (Cohen et al., 2021; Liu et al., 2023). For example, utilizing English-French bilinguals as participants and alphabetic languages (e.g., English and French) as experimental materials, neuroimaging studies that used a millimeter-scale 7-T high-resolution scanner and univariate analysis (i.e., activation analysis) showed that, whether for English or French, the posterior subregions were sensitive to sublexical visual features, while the anterior subregions were sensitive to high-level lexical information (Zhan et al., 2023). Consistently, studies using multivariate analysis (e.g., representational similarity analysis [RSA]) also revealed a similar hierarchical gradient (Fischer-Baum et al., 2017; Taylor et al., 2019). These findings suggest that the posterior and anterior VWFA show distinct functions in word reading.

In addition to the functional specialization of VWFA subregions, recent research has found that the connectivity patterns between VWFA subregions and the language networks appear to align with their respective functions (Caffarra et al., 2021; Chauhan et al., 2024). Specifically, they identified two VWFAs (i.e., VWFA-1 and VWFA-2) in lvOT. Using structural connectivity analysis, studies found that VWFA-2 (the anterior VWFA) connects to language regions by the arcuate fasciculus, while VWFA-1 (the posterior VWFA) connects to visual regions by the vertical occipital fasciculus (Takemura et al., 2016; Yeatman & White, 2021). Similarly, studies using functional connectivity analysis revealed a robust connectivity between VWFA-2 and language processing regions in the frontal and lateral parietal lobes, and a similar robust connectivity between VWFA-1 and the visual processing regions in the ventral occipital cortex (Li et al., 2024; Yablonski et al., 2024).

Integrating the results of neural activity and connectivity patterns, it appears to support the connectivity-biased hypothesis, which posits that the functions were regulated by the local generalized neurocomputation and the varying connectivity patterns (De¸bska et al., 2023; Humphreys et al., 2020, 2021). From this perspective, the function of VWFA is reflected not only in its local activation, but also in its structural and functional connectivity patterns with high-level reading-related regions. In support of this hypothesis, recent research has found that the local activation of the VWFA and its functional and effective connectivity with other regions jointly contribute to lexical processing (Li et al., 2024). However, whether the functional specialization of VWFA subregions originates from the connectivity remains unclear for at least three reasons. First, previous studies mainly explored the functional role of VWFAs in alphabetic languages, which have relatively obvious grapheme-to-phoneme correspondence (GPC) rules (Fischer-Baum et al., 2017; Lo & Yeh, 2018). Therefore, it is difficult to disentangle the contributions of orthography and phonology to neural activations in lvOT (Glezer et al., 2016; Gu et al., 2024; Rumsey et al., 1997). In contrast, logographic languages, such as Chinese characters, are constructed mostly by a phonetic radical and a semantic radical, deviating from the GPC rules (Guo et al., 2022; Yu & Reichle, 2017). Due to the imperfect correspondence between the orthographic and phonological information, Chinese characters are more suitable for addressing the question of disentangling different lexical representations in VWFA. Although several studies used ideographic characters that deviated from the GPC rules as materials combined with RSA to explore the functional role of VWFAs, they adopted a relatively small number of materials (Liu et al., 2023; Zhao et al., 2017), resulting in low ecological validity. Therefore, it is necessary to use a large number of ideographic characters (e.g., Chinese characters) to disentangle different lexical representations in VWFAs. Second, most studies examined the functional roles and connectivity patterns using an unimodal imaging approach (e.g., task-state fMRI, resting-state fMRI, or diffusion MRI), which only provided limited insight into the neural mechanism. Therefore, multimodal neuroimaging methods are necessary in a single study to comprehensively investigate the neural activities and connectivity patterns of VWFA subregions. Finally, previous studies have found that the processing demands affect the neural mechanisms of word reading (Branzi et al., 2022; Price & Devlin, 2011). Therefore, it is necessary to adopt multiple reading tasks to explore the modulatory effects of processing demands on the neural activity and connectivity of VWFAs simultaneously.

To address the above issues, the present study adopted multiple neuroimaging techniques (dMRI, resting-state fMRI, and task-state fMRI) to investigate the neural representations and brain circuitry of VWFAs during Chinese reading in two experiments. Experiment 1 examined the resting-state functional connectivity and the structural connectivity between two VWFAs (i.e., VWFA-1 and VWFA-2) and the functionally localized language networks (i.e., orthographic, phonological, and semantic networks defined by the radical judgment, rhyme judgment, and semantic judgment task, respectively). Experiment 2 used two reading tasks (i.e., structure and familiarity judgment tasks) combined with RSA to explore the neural representations of lexical information and the modulatory effects of processing demands.

## MATERIALS AND METHODS

### Experiment 1

Experiment 1 adopted the classical language network localizer tasks (i.e., the radical judgment, rhyme judgment, and semantic judgment tasks) to identify the corresponding language networks (i.e., orthographic, phonological, and semantic networks), respectively (Li et al., 2025; Liu et al., 2022). Then, the structural and functional connectivity patterns between two VWFAs (i.e., VWFA-1 and VWFA-2) and the language networks were examined. Based on previous findings (Caffarra et al., 2021; Li et al., 2024), we expected that VWFA-1 (the posterior VWFA) would have stronger structural and functional connectivity with the orthographic network, while VWFA-2 (the anterior VWFA) would have stronger connectivity with the phonological and semantic networks.

#### Materials and methods

##### Participants.

One hundred and eleven Chinese college students (63 females, mean age = 21.29 years, *SD* = 2.31 years) participated in Experiment 1. Among them, 32 participants completed the language network localizer tasks (i.e., the radical judgment, rhyme judgment, and semantic judgment tasks) to localize the orthographic, phonological, and semantic networks of Chinese characters. This sample size was determined for two reasons. First, we utilized G*Power (G*Power 3.1.9.7, effect size = 0.3, power = 0.8) to estimate the appropriate sample size needed for our design (Cohen, 1977, 1992; Faul et al., 2009). The expected sample size is 20 participants. Second, the previous fMRI studies that used the localizer task to define reading-related networks typically recruited approximately 30 participants (Graves et al., 2023; Gu et al., 2024; Liu et al., 2022), indicating that a sample size of 30 is sufficient for reliably localizing the reading-related networks. A total of 111 participants completed both the resting-state fMRI and dMRI scans. Six participants' DTI data were excluded from the analysis due to inconsistent parameters, resulting in 105 participants in the DTI analysis. All participants were right-handed with normal hearing and vision (Snyder & Harris, 1993). All participants provided written informed consent before the experiment. Institutional Review Board has approved the ethical procedures.

##### Materials.

**Figure 1.**
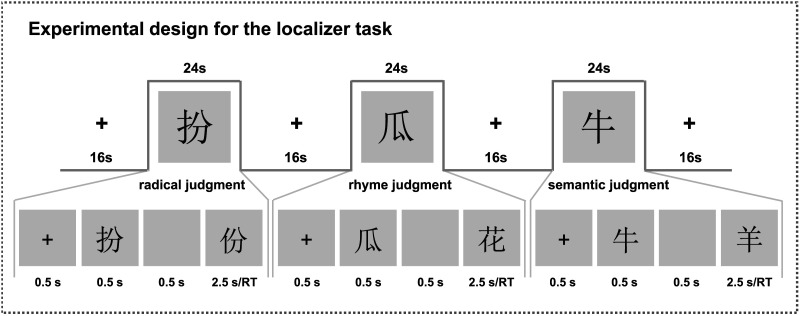
Experimental design of the language network localizer task. During the language network localizer task, participants were instructed to judge whether the two characters shared the same radical (the radical judgment task), phonologically rhymed (the rhyme judgment task), or semantically related (the semantic judgment task).

The language network localizer task (LN localizer task) comprised 108 pairs of Chinese characters (Figure 1). To improve the ecological validity, these Chinese characters included nouns, verbs, adjectives, and prepositions. The characters were of medium to high frequency (102.82/million words; Cai & Brysbaert, 2010), and the mean number of strokes was 6.97. Chinese characters were then assigned to 3 subtasks, namely the radical judgment task, the rhyme judgment task, and the semantic judgment task. For the radical judgment task, 18 pairs of Chinese characters shared the same radical (e.g., the Chinese characters “扮” and “份” shared the radical “分”), and the other 18 pairs did not. For the rhyme judgment task, 18 pairs of Chinese characters rhymed (e.g., the Chinese characters “瓜”/gua/ and “花/hua/” rhymed with /ua/), and the other 18 pairs did not. For the semantic judgment task, 18 pairs of Chinese characters were semantically related (e.g., the Chinese characters “牛”/cattle/ and “羊”/sheep/), and 18 pairs were semantically unrelated. Notably, to minimize mutual interference among orthographic, phonological, and semantic information, character pairs in the radical judgment task do not rhyme phonologically and are not semantically related. Character pairs in the rhyme judgment task share no radicals and are not semantically related. Character pairs in the semantic judgment task share no radicals and do not rhyme phonologically.

##### fMRI task.

Before fMRI scanning, participants completed a practice to understand the procedure of the experiment. All experimental materials were presented by Psychtoolbox-3 in MATLAB (www.psychtoolbox.org).

fMRI scans included 3 runs of the LN localizer task with block design (Figure 1). Each run comprised 6 blocks, with 2 blocks per task (i.e., the radical judgment, rhyme judgment, and semantic judgment tasks). Each task block lasted for 24 s and was separated by 16 s fixation blocks. The task blocks were separated by 16 s fixation blocks, and the order of tasks was counterbalanced across participants. Participants were required to respond to the stimuli based on the instructions of each task. Each task block contained 6 trials. Each task block lasted for 24 s and contained 6 trials. Each trial began with a 0.5 s fixation, followed by two stimuli presented sequentially. The first character was displayed for 0.5 s, followed by a 0.5 s interval. The second character was displayed for at most 2.5 s and disappeared after the participant responded. In the radical judgment task, participants were instructed to judge whether the two characters shared the same radical (e.g., “扮” and “份” shared the same radical “分”). In the rhyme judgment task, participants were instructed to judge whether the two characters rhymed (e.g., “花 /hua/” rhymes with “瓜 /gua/”). In the semantic judgment task, participants were instructed to judge whether the two characters were semantically related (e.g., “牛 /cow/” is semantically related to “羊 /sheep/”). Across all runs, the total number of trials per task was 36 (3 runs × 2 blocks × 6 trials). The buttons were balanced across participants.

##### MRI scanning protocols.

MRI images were collected by a 3T Siemens Prisma scanner at the [redacted]. Functional images using the T2-weighted gradient echo planar imaging sequence: TR = 2,000 ms, TE = 30 ms, flip angle = 90°, FOV = 224 mm × 224 mm, image matrix = 112 × 112, voxel size = 2 mm × 2 mm × 3 mm. Fifty-eight 2-mm-thick axial slices were obtained to fully map the brain. During resting-state fMRI scanning, participants were asked to view the fixation on the screen. A total of 240 volumes of resting-state fMRI were acquired for each participant.

Structural images were obtained by a T1-weighted, MPRAGE sequence with parameters: TR = 2,530 ms, TE = 1.94 ms, flip angle = 7°, 176 slices with 0.5-mm thickness, and voxel size = 1 mm × 0.5 mm × 0.5 mm.

Diffusion images were acquired with the parameters: TR = 8300 ms, TE = 84 ms, FOV = 256 mm × 256 mm, matrix size = 128 × 128, number of slices = 64, slice thickness = 2 mm, and voxel size = 2 mm × 2 mm × 2 mm. Each diffusion data has 64 gradient directions with a b-value of 700 s/mm^2^ and 10 b0-volumes.

#### MRI data analysis

##### Activation analysis in the language network localizer task.

MRI data were preprocessed using FEAT 6.00 in FSL (www.fmrib.ox.ac.uk/fsl). The functional images underwent motion correction and were stripped of non-brain tissue (Jenkinson et al., 2002; Smith, 2002). Then, they were temporally filtered and registered to standard Montreal Neurological Institute (MNI) space (Andersson et al., 2007; Jenkinson & Smith, 2001). The functional images were spatially smoothed for univariate analysis using a Gaussian kernel of 5 mm full-width-half-maximum (FWHM).

We performed a whole-brain activation analysis to localize the orthographic, phonological, and semantic networks. At the first level of activation analysis, we modeled the preprocessed data by the general linear model (GLM) for each task condition in each run of each participant. The onsets and durations of the events were convolved with the double-gamma hemodynamic response function (HRF) to generate the regressors used in the GLM. Six motion parameters and temporal derivatives were included as covariates to reduce the impact of motion artifacts. Three contrast images (radical judgment > rhyme + semantic judgment, rhyme judgment > radical + semantic judgment, and semantic judgment > rhyme + radical judgment) were computed separately for each run and for each participant to determine the orthographic, phonological, and semantic networks. Then, a second-level analysis was conducted by using a fixed-effects model, averaging the activation of the orthographic, phonological, and semantic networks across the 3 runs separately to obtain the activation of each network for each participant. Finally, a random-effects model was used to obtain group activations (Beckmann et al., 2003; Woolrich, 2008). According to previous studies (Gu et al., 2024; Zhang et al., 2024), all group images of activation results were thresholded by the voxel-wise cutoff at Z > 3.1 (i.e., one-tailed test with *p* < 0.005) and a cluster probability of *p* < 0.05, corrected for whole-brain multiple comparisons using the Gaussian random field theory (GRF; Worsley, 2001).

The activation results at the group level were binarized to obtain the final orthographic, phonological, and semantic networks. To avoid the interference of autocorrelation on the connectivity results, we excluded the overlapping regions of lvOT from the orthographic network using the fslmaths function within FSL (www.fmrib.ox.ac.uk/fsl).

##### Resting-state functional connectivity analysis.

The resting-state functional connectivity (rsFC) analysis was performed using the DPARSF (Data Processing Assistant for Resting-State fMRI, https://rfmri.org/DPARSF). Following previous studies (Wang et al., 2015; Yan et al., 2016), resting-state fMRI images were first preprocessed using the following steps: removal of the first ten functional volumes to allow for T1-equilibration effects, slice timing correction, head motion correction, normalization of the functional images into the MNI space, spatial smoothing with a 4 mm FWHM Gaussian kernel, a band-pass filter (0.01–0.08 Hz) was applied to reduce low-frequency drift and high-frequency noise, 9 nuisance covariates (6 head motion parameters and 3 regressors corresponding to the whole brain, white matter, and cerebrospinal fluid signal) were regressed out to control for physiological effects and head motion. We defined two VWFAs in lvOT based on the MNI coordinates reported in previous research (White et al., 2023). To improve the signal-to-noise ratio and contribute to more stable results, we defined these regions of interest (ROIs) as 9-mm-radius spherical regions in line with previous studies (Liu et al., 2024; Ruan et al., 2025; Figure 3A). Specifically, the MNI coordinates of the posterior VWFA were −43, −61, −10 (VWFA-1), and those of the anterior VWFA were −46, −45, −18 (VWFA-2). Then, the resting-state time series of all voxels within each VWFA was extracted and averaged to obtain the resting-state time course for each VWFA.

We first conducted a seed-based whole-brain functional connectivity analysis. Specifically, we conducted a Pearson correlation analysis on the time series of two VWFAs and those of all other voxels in the whole brain. All correlation coefficients were transformed into Fisher's Z-scores. Then, a paired-sample *t* test was performed between the correlation maps of VWFA-1 and VWFA-2 to compare the FC differences between the posterior and anterior VWFA subregions. To further explore the degree of overlap between the orthographic, phonological, and semantic networks and the whole-brain FC map from VWFA-1 and VWFA-2, we calculated the number and proportion of overlapping voxels in different networks, respectively. Specifically, we first binarized the whole-brain FC maps from VWFA-1 and VWFA-2 with a threshold at Z > 3.1, respectively. Subsequently, we multiplied the binarized functional connectivity results and the binarized orthographic, phonological, and semantic networks, respectively, to yield the binarized connectivity results between VWFA-1/VWFA-2 and each voxel within the orthographic, phonological, and semantic networks. Then, we used the fslstats function within FSL (www.fmrib.ox.ac.uk/fsl) to calculate the number of overlapping voxels between the whole-brain FC maps from VWFA-1 and VWFA-2 and the orthographic, phonological, and semantic networks, respectively. The proportion of overlapping voxels in different language networks was calculated as the number of overlapping voxels within the orthographic, phonological, and semantic networks divided by the total number of voxels showing robust functional connectivity between the whole brain and VWFA-1/VWFA-2 (DeMayo et al., 2023; Peer et al., 2021).

To further explore the FC differences between the two VWFAs and the language networks, we conducted the ROI-to-ROI analysis to quantify the connectivity strength between VWFAs. Specifically, we conducted a Pearson correlation analysis on the time series of two VWFAs and those of the orthographic, phonological, and semantic networks. These correlation coefficients were transformed into Fisher's Z-scores, which are unitless. Then, a two-way repeated measures analysis of variance (ANOVA) was conducted on the connectivity strengths between two VWFAs and the networks (the orthographic, phonological, and semantic network).

##### Structural connectivity analysis.

The dMRI data were preprocessed and analyzed by probabilistic fiber tracking with the FDT 5.0 toolbox in FSL (https://fsl.fmrib.ox.ac.uk/fsl/fslwiki/FDT). Specifically, we first extracted all b0 images and mapped the Diffusion Tensor Imaging (DTI) data onto the b0 images by the 12-parameter affine transformation method in the FDT toolbox. Next, the TOPUP tool was used for estimating and correcting susceptibility-induced distortions, and the EDDY tool was used to correct for eddy current-induced distortions and participant movements. Finally, the corrected images were re-registered back to the individual space for subsequent analysis.

The BedpostX tool in FSL was used to perform Bayesian estimation of the diffusion parameters. The “ball and stick” multicompartment decomposition model was adopted to model the white matter fiber directions and crossing fibers, and a distribution model for the diffusion parameters in each voxel was established (Behrens et al., 2003). Then, the individual-level outputs were segmented by the Probtrackx tool to generate connectivity maps. The specific parameters were: the tracked streamlines = 5,000; the maximum number of steps = 2,000, step size = 0.5, and turning angle threshold = 0.2. Subsequently, probabilistic fiber tracking between two VWFAs and the orthographic, phonological, and semantic networks was carried out, and reverse-direction tracking was also performed. The mean of the results from the two-way tracking was taken as the final number of fiber connections. Finally, to minimize the potential confounding effects of the volume size (i.e., structural connectivity may increase with the volume of each network), the strength of the structural connectivity was divided by the number of voxels in each network to normalize the strength of the connectivity, which is also unitless.

Subsequently, a two-way ANOVA was conducted on the structural connectivity strengths between two VWFAs and the networks (the orthographic, phonological, and semantic network).

#### Results

##### Behavioral results.

**Figure 2.**
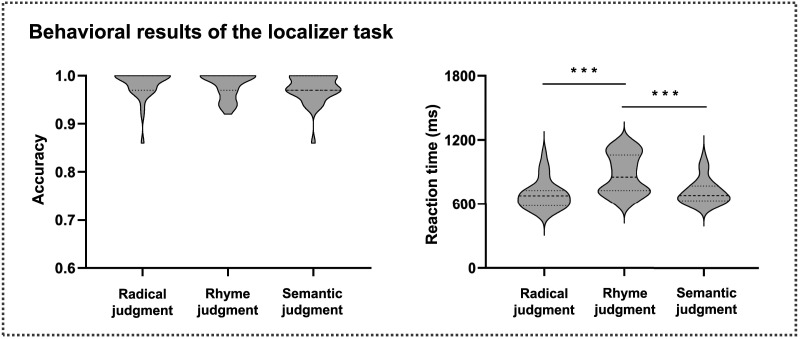
The behavioral results of the language network localizer task. During the language network localizer task, participants were instructed to judge whether the two characters shared the same radical (the radical judgment task), phonologically rhymed (the rhyme judgment task), or semantically related (the semantic judgment task). Violin plots indicate the ranges of accuracy and reaction time for the radical judgment, rhyme judgment, and semantic judgment tasks. ***p < 0.001.

For the LN localizer task, the mean reaction time (RT) was 689.19 ms (*SD* = 136.47 ms), 879.37 ms (*SD* = 188.34 ms), and 716.42 ms (*SD* = 117.74 ms) for the radical judgment task, the rhyme judgment task, and the semantic judgment task, which falls within the range of mean RTs reported in previous studies that employed the same experimental paradigm (Liu et al., 2022; Tao et al., 2024). One-way ANOVA showed a significant main effect of RT across tasks (*F*_2, 62_ = 34.332, *p* < 0.001). Post hoc comparisons revealed that the RT in the rhyme judgment task was longer than that in the radical judgment task (*p* < 0.001) and the semantic judgment task (*p* < 0.001), whereas no significant difference in RT was observed between the latter two (*p* = 0.126). The mean accuracy was 0.98 (*SD* = 0.03), 0.98 (*SD* = 0.03), and 0.97 (*SD* = 0.03) for the radical judgment task, the rhyme judgment task, and the semantic judgment task, indicating that participants were attentive during the task (Figure 2). One-way ANOVA showed no significant difference of accuracy across tasks (*F*_2, 62_ = 1.669, *p* = 0.197).

##### Activation results in the language network localizer task.

Overall, Chinese characters elicited stronger activations in reading-related regions than baseline, which is consistent with previous research (Qu et al., 2022; Zhan et al., 2023).

Subsequently, we defined the orthographic, phonological, and semantic networks by contrasting activation patterns under different conditions in the LN localizer task (Figure 4A). Specifically, the orthographic network was defined based on the greater activation for the radical judgment condition relative to the rhyme and semantic judgment conditions, which included the posterior parietal lobe, inferior temporal lobe, and occipital lobe (Supplementary Figure 1A; Supporting Information can be found at https://doi.org/10.1162/NOL.a.225). The phonological network was defined based on the greater activation for the rhyme judgment condition relative to the radical and semantic judgment conditions, which included the middle frontal gyrus, inferior frontal gyrus (IFG), precentral gyrus, and parts of the temporoparietal cortex (Supplementary Figure 1B). The semantic network was defined based on the greater activation for the semantic judgment condition relative to the rhyme and radical judgment conditions, which included the superior frontal gyrus (SFG), middle frontal gyrus, IFG, middle temporal gyrus, and posterior temporoparietal cortex (Supplementary Figure 1C).

To avoid potential confounding effects of RT differences, we incorporated each participant's RT under different tasks as a covariate to further validate our localization of the language networks. Results showed high overlap between language networks defined after controlling for RT and the original networks (89% for the orthographic network, 80% for the phonological network, and 90% for the semantic network), indicating that task-related RT differences did not significantly impact the localization of language networks.

##### Resting-state functional connectivity results.

**Figure 3.**
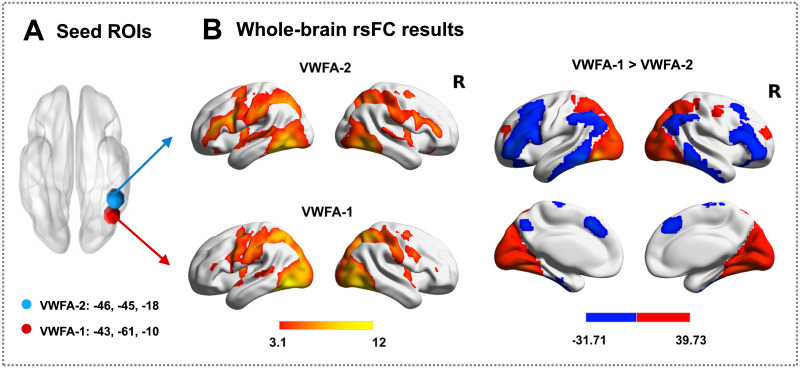
The ROIs defined based on previous research (A) and the rsFC results between the whole-brain and visual word form areas (VWFAs; B). The Montreal Neurological Institute coordinates of VWFA-1 were −43, −61, −10 (VWFA-1), and those of VWFA-2 were −46, −45, −18 (VWFA-2) based on previous research (White et al., 2023). rsFC = resting-state functional connectivity. R = right hemisphere.

The seed-based whole-brain functional connectivity analysis showed that VWFA-1 revealed robust FC to the temporoparietal and occipital cortex, while VWFA-2 revealed robust FC to the IFG, temporoparietal cortex, and occipital cortex. A direct comparison of the whole-brain FC map between two VWFAs showed that VWFA-1 had stronger FC to the superior parietal gyrus, vOT, and occipital pole. In contrast, VWFA-2 had stronger FC to the middle SFG, opercular and triangular parts of the IFG, superior temporal, and middle temporal gyrus (Figure 3B).

**Figure 4.**
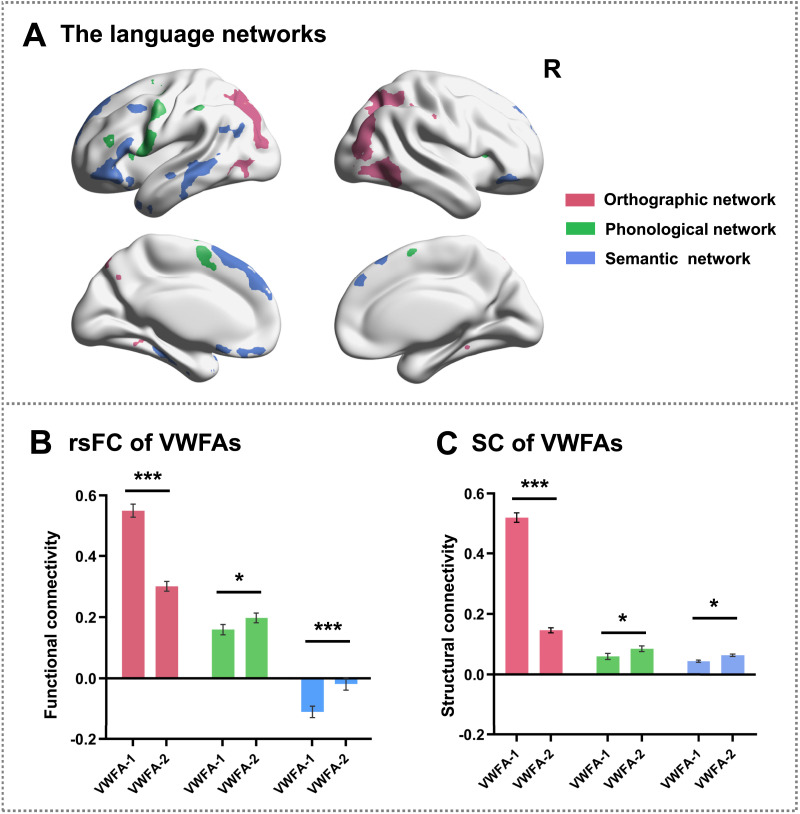
The language networks defined by the language network localizer task (A). The resting-state functional connectivity (B) and the structural connectivity (C) results between the two visual word form areas (VWFAs) and the language networks. The orthographic network (pinkish red) was defined by the contrast of radical judgment > rhyme + semantic judgment task, the phonological network (green) was defined by the contrast of rhyme judgment > radical + semantic judgment task, and the semantic network (blue) was defined by the contrast of semantic judgment > rhyme + radical judgment task. The histograms show the strength of the connectivity between the VWFAs and orthographic (pinkish red), phonological (green), and semantic (blue) networks. Error bars represent the standard error of the mean. ***p < 0.001. *p < 0.05. R = right. rsFC = resting-state functional connectivity. SC = structural connectivity.

To further explore the functional connectivity differences between two VWFAs and the language networks, a two-way ANOVA was conducted on the connectivity strengths between different VWFAs (VWFA-1 and VWFA-2) and the networks (the orthographic, phonological, and semantic network; Figure 4B). The results showed a significant main effect of the networks (*F*_2,220_ = 208.222, *p* < 0.001, η_p_^2^ = 0.652). Post hoc comparisons revealed that VWFAs had stronger FC to the orthographic network than the phonological and semantic networks, while they had stronger FC to the phonological than the semantic network (*p*s < 0.001, false discovery rate [FDR] corrected). We also found a main effect of the VWFAs (*F*_1,110_ = 19.187, *p* < 0.001, η_p_^2^ = 0.147). Specifically, VWFA-1 exhibited more robust FC with language networks than VWFA-2 (*p* < 0.001). Furthermore, the network-by-VWFA interaction was significant (*F*_2,220_ = 86.212, *p* < 0.001, η_p_^2^ = 0.437). Simple-effects analysis showed that the FC to the orthographic network from VWFA-1 was significantly stronger than that of VWFA-2, while the FC to the phonological network and the semantic network from VWFA-2 were significantly stronger than those of VWFA-1 (*p*s < 0.05, FDR corrected).

##### Structural connectivity results.

To further explore the structural connectivity differences between the language networks and the VWFAs, a 3 (networks: orthographic, phonological, and semantic networks) × 2 (VWFAs: VWFA-1 and VWFA-2) two-way ANOVA was conducted (Figure 4C). Results showed a significant main effect of the networks (*F*_2,208_ = 487.065, *p* < 0.001, η_p_^2^ = 0.824). Post hoc comparisons revealed that VWFAs had stronger structural connectivity to the orthographic network than the phonological and semantic networks (*p* < 0.001, FDR corrected), while they had stronger FC to the phonological than the semantic network (*p* < 0.05, FDR corrected). We also found a significant main effect of the VWFAs (*F*_1,104_ = 258.737, *p* < 0.001, η_p_^2^ = 0.713). Specifically, VWFA-1 exhibited more robust structural connectivity with language networks than VWFA-2 (*p* < 0.001). More importantly, we found a significant network-by-VWFA interaction (*F*_2,208_ = 296.785, *p* < 0.001, η_p_^2^ = 0.741). Further simple-effects analysis showed that the structural connectivity between the orthographic network and VWFA-1 was significantly stronger than that of VWFA-2, while the structural connectivity to the phonological and semantic network from VWFA-2 was significantly stronger than that of VWFA-1 (*p*s < 0.05, FDR corrected).

To further validate the results of the VWFA defined at the group level, we further calculated the structural and functional connectivity between the language networks and the VWFAs defined at the individual level. Results showed that the patterns of both functional and structural connectivity between the language networks and the VWFAs defined at the individual level were consistent with those observed at the group level (Supplementary Materials B). Notably, due to the VWFAs defined at the individual level containing fewer voxels compared to those defined at the group level, the strength of functional and structural connectivity between the language networks and the VWFAs defined at the individual level was lower than that observed at the group level.

It is worth mentioning that when we validate the structural and functional connectivity results between the language networks and the individual-level VWFAs, we employed different thresholds across participants to ensure a sufficient number of surviving voxels following previous studies (Glezer et al., 2016; Gu et al., 2023). However, when a consistent and more stringent threshold (Z = 3.1) was used, the majority of participants failed to define the VWFAs at the individual level (i.e., 58 out of 77 participants for VWFA-1 and 66 out of 77 participants for VWFA-2). These results are consistent with previous research that, with equal thresholds, the VWFA often fails to appear where expected for many participants (Neudorf et al., 2022).

Combining resting-state fMRI and dMRI techniques, Experiment 1 explored the structural and functional connectivity patterns between the two VWFAs (i.e., VWFA-1 and VWFA-2) and the language networks (i.e., orthographic, phonological, and semantic networks). Consistent with previous research (Li et al., 2024; Yablonski et al., 2024), VWFA-1 (the posterior VWFA) exhibited robust structural and functional connectivity to the orthographic network, while VWFA-2 (the anterior VWFA) exhibited robust structural and functional connectivity to the phonological and semantic networks. These converging results of both structural and functional connectivity provided multimodal neuroimaging evidence for the connectivity patterns of VWFA subregions. As discussed in the Introduction section, the connectivity-biased computation hypothesis posits that the functions were regulated by the local generalized neurocomputation and the varying connectivity patterns. Therefore, Experiment 2 utilized structure and familiarity judgment tasks combined with RSA to explore the neural representations in two VWFAs and the modulatory effects of processing demands.

### Experiment 2

Experiment 2 used representational similarity analysis (RSA) to explore the functional role of two VWFAs. In addition, we examined the modulatory effects of processing demands by comparing the neural representations in the structural judgment task and the familiarity judgment task. Based on previous findings of the functional gradient in lvOT (Krafnick et al., 2016; Zhan et al., 2023), we expected that VWFA-1 (the posterior VWFA) would represent visual information while VWFA-2 (the anterior VWFA) would represent high-level semantic information. In addition, processing demands modulate the representational patterns of VWFAs.

#### Materials and methods

##### Participants.

Thirty-two college students (17 females, mean age = 21.89 years, *SD* = 1.95 years old) were recruited in Experiment 2. They were right-handed with normal hearing and vision. All participants provided written informed consent before the experiment.

##### Materials.

**Figure 5.**
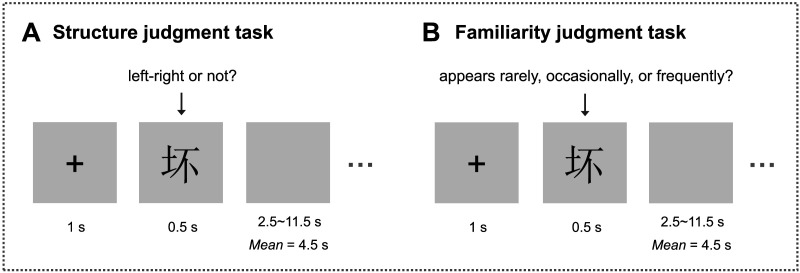
Experimental design of the structure (A) and familiarity judgment task (B). During the structure judgment tasks, participants were asked to judge the structure of the Chinese character (left-right compound character or not). During the familiarity judgment tasks, participants were asked to rate the frequency of the content represented by Chinese characters in their daily lives (appears rarely, appears occasionally, or appears frequently).

A total of 198 common Chinese characters were used for the Chinese character structure and familiarity judgment tasks (Figure 5). Among these characters, 99 were left-right compound characters, 78 were top-bottom compound characters, and 21 were other structures. All the Chinese characters were high-frequency characters (210.5/million words; Cai & Brysbaert, 2010), and the mean number of strokes was 10.09 (*SD* = 2.81). To ensure that the materials were selected with a high level of ecological validity, the present study balanced the part of speech of 198 Chinese characters. All the materials were crafted into images with black characters on a gray background, and the resolution of these images was 302 × 302 pixels.

##### Visual, phonological, and semantic dissimilarity matrices.

The present study constructed behavioral representational dissimilarity matrices (DSM) for 198 Chinese characters in three types of stimulus features (i.e., visual DSM, phonology DSM, and semantics DSM), respectively, enabling subsequent representational similarity analyses with brain neural DSM.

Specifically, the visual representation of each character was constructed by converting each Chinese character into pictorial form (604 × 604 pixels) and performing binarization processing, with 0 representing background pixels and 1 representing image pixels. Each binary silhouette image was then converted to a pixel vector. The visual DSM was calculated as 1 minus the Pearson correlation (cosine similarity) for all pairs of Chinese characters (198 × 198 = 39,204 pairs).

For the phonological DSM, each character was first converted into pinyin (e.g., “学” was converted into “xue”) based on segment-and-feature systems, and then was decomposed into 23 onsets, 34 vowels, 5 tones, and an additional unit (used to represent Chinese characters without onsets; Perfetti et al., 2005). A 63-bit binary phonological feature value was constructed based on the inclusion relationship (assigned a value of 1 if the syllable was included, and assigned a value of 0 if not). The phonological DSM was calculated as 1 minus the Pearson correlation for all pairs of Chinese characters.

The semantic feature dissimilarity matrix was constructed following an open-source word vector training tool, the word2vec method (https://code.google.com/p/word2vec/). Based on a large-scale corpus, the word vectors of the selected Chinese characters were extracted, and the cosine similarity between each pair of these Chinese characters was calculated. The semantic DSM was calculated as 1 minus the Pearson correlation for all pairs of Chinese characters. All dissimilarity coefficients were converted to Fisher's Z-scores. Consequently, 198 × 198 DSMs of visual, phonology, and semantics were constructed.

##### fMRI task.

The fMRI scans included 6 runs of structure judgment tasks and 6 runs of familiarity judgment tasks with event-related design (Figure 5). Each run contained 66 Chinese character stimuli, which were presented in a pseudo-random order. Each Chinese character was repeated twice in each task, the first presentation occurred in the first 3 runs, and the second presentation was in the last 3 runs. There was an interval of at least 2 runs between the repeated presentations of the same Chinese character to avoid interference from identical materials. Each trial began with a fixation for 1 s, followed by a character for 0.5 s. For the structure judgment tasks, participants were asked to judge the structure of the Chinese character (left-right compound character or not). For the familiarity judgment tasks, participants were asked to rate the frequency of the content represented by Chinese characters in their daily lives (appears rarely, appears occasionally, or appears frequently). The experimental sequence and intervals were optimized by OPTSEQ2 (https://surfer.nmr.mgh.harvard.edu/optseq/; Dale, 1999). The buttons were balanced across participants. After the stimulus disappeared, a blank screen was presented, varying randomly from 2.5 to 11.5 s (*M* = 4.5 s).

#### MRI data analysis

##### Activation analysis in the structure and familiarity judgment tasks.

Structural and functional MRI images were collected with the parameters of Experiment 1. Image preprocessing in the structure and familiarity judgment tasks was the same as in Experiment 1. For RSA, no spatial smoothing was applied to preserve the fine-grained spatial patterns of neural activity (Li et al., 2023; Liuzzi et al., 2023).

We performed whole-brain activation analysis in the structure and familiarity judgment tasks, respectively. First, we modeled the preprocessed data by GLM for each run in each task. The onsets and durations of the Chinese characters were convolved with the double-gamma HRF to generate the regressors used in the GLM. The blank screen and fixation served as the baseline. Then, we used the fixed-effects model to obtain the mean activation of each task for each participant. Finally, we used the random-effects model to obtain group activation of each task.

##### Representational similarity analysis.

Region-of-interest-based (ROI-based) RSA was performed on the data of the structure judgment and familiarity judgment task to decode the visual, phonological, and semantic information underlying the neural representation of the two VWFAs. Consistent with Experiment 1, we defined VWFA-1 (the posterior VWFA) and VWFA-2 (the anterior VWFA) as two ROIs following previous studies (White et al., 2023). In addition, due to the crucial involvement of the occipital cortex in visual processing, we defined the occipital cortex as an ROI based on the Harvard-Oxford atlas with a 25% threshold to examine the effectiveness of our experiment. First, we modeled unsmoothed data for each run in each task to obtain the trial-level neural response patterns (i.e., beta maps). Each Chinese character was modeled as a separate regressor and convolved with a canonical double-gamma HRF. Subsequently, the beta map of each Chinese character was obtained by ordinary least squares estimation and ridge regression (Qu et al., 2022; Xue et al., 2010). The beta maps of two repetitions of each character in each task were averaged to obtain the final neural activity.

Next, we extracted voxel-wise response patterns of 198 characters in each ROI and each task. Dissimilarities in response patterns between every pair of words were estimated by using the correlation distances (1 - Pearson's correlation). This step resulted in a 198 × 198 neural DSM for each participant and each task. These neural matrices were then Fisher-transformed.

Subsequently, we extracted the lower triangular elements of each 198 × 198 DSM (neural, visual, phonological, and semantic DSM) and flattened them into a one-dimensional vector. The Spearman correlation analysis was conducted between the flattened neural DSMs and the flattened visual, phonological, and semantic DSMs. These correlation coefficients were transformed into Fisher's Z-scores. Finally, a one-tailed statistical test was used to identify whether the correlation was higher than zero in each ROI. To prevent an increase in false positive rates, we used a 5,000-time nonparametric permutation test with a 95% confidence interval to determine the significance level following previous studies (Liu et al., 2024; Pacheco-Estefan et al., 2024; Zhao et al., 2017). Specifically, the correlation between the neural DSM and the lexical feature DSMs was regarded as the true value. Then we randomly shuffled three lexical feature DSMs separately and calculated Spearman's correlation between the neural DSM and each shuffled lexical feature DSM, to derive a null correlation value for each permutation. All correlation coefficients were converted into Fisher's Z-scores. We executed this process 5,000 times and compared the observed Z-value with the null distribution derived from 5,000 permutations. The significance of the true Z-value was determined using the equation: *p* = ((the number of permutated Z-values > the true Z-value) + 1) / 5,001.

#### Results

##### Behavioral results.

**Figure 6.**
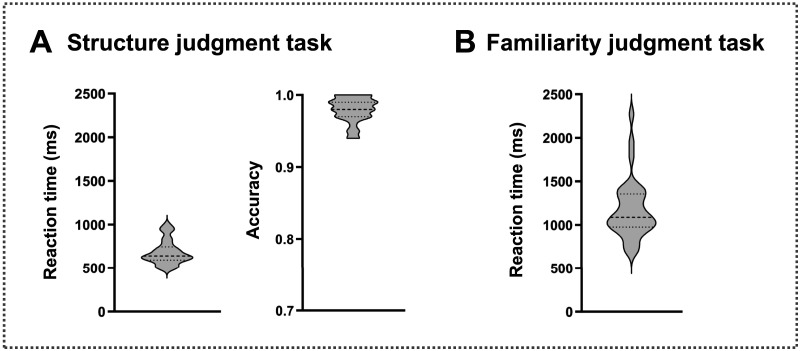
The behavioral results of the structure judgment task (A) and the familiarity judgment task (B). During the structure judgment tasks, participants were asked to judge the structure of the Chinese character (left-right compound character or not). During the familiarity judgment tasks, participants were asked to rate the frequency of the content represented by Chinese characters in their daily lives (appears rarely, appears occasionally, or appears frequently). Violin plots indicate the ranges of accuracy and reaction time for the structure and familiarity judgment tasks.

The response time was 679.79 ms (*SD* = 138.33 ms) for the structure judgment task and 1,176.70 ms (*SD* = 343.70 ms) for the familiarity judgment task, which is comparable to the RTs reported in previous studies by similar paradigms (Liu et al., 2022; Zhang et al., 2020). Due to the fact that the familiarity judgment task involves participants' subjective judgments, we did not calculate the accuracy. The accuracy was 0.98 (*SD* = 0.02) for the structure judgment task, indicating that the participants as a whole were earnest and the experimental design was effective (Figure 6).

##### Activation results in the structure and familiarity judgment tasks.

Consistent with previous studies, Chinese characters elicited stronger activations in the reading-related regions than baseline for both structure and familiarity judgment tasks, including bilateral prefrontal cortex, temporoparietal cortex, and occipitotemporal cortex (Li et al., 2024; Qu et al., 2022; Supplementary Figure 4).

##### Visual, phonological, and semantic representations.

To examine the effectiveness of the present experiment, we first explored the correlations between the neural DSMs and the visual DSM in the occipital cortex (Supplementary Figure 5). Results showed that the visual information was represented in the occipital cortex in both the structure and familiarity judgment tasks, indicating that all the participants were earnest and the experimental design was effective.

**Figure 7.**
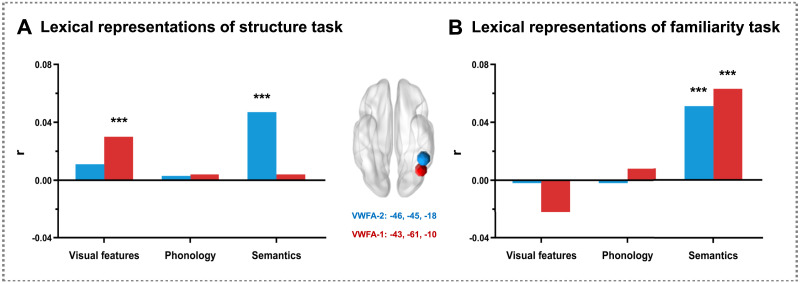
The representation results of the lexical information in the structure judgment task (A) and the familiarity judgment task (B). The Montreal Neurological Institute coordinates of visual word form area (VWFA)–1 were −43, −61, −10, and those of VWFA-2 were −46, −45, −18. Spearman correlations in two ROIs were calculated between the neural DSMs and the visual, phonological, and semantic DSMs, respectively. The correlation coefficients were Fisher Z-transformed. A 5,000-permutation test was performed and a 95% confidence interval was used. The *x*-axis represents the z value after Fisher's Z transform. ***p < 0.001.

To examine the neural representation of visual, phonological, and semantic information in VWFA subregions, we calculated the correlations between neural DSMs and the visual, phonological, and semantic DSMs in two VWFAs (Figure 7 and Supplementary Figure 6). For the structure judgment task, the results of the permutation test showed that the visual information was represented in VWFA-1, while the semantic information was represented in VWFA-2 (Figure 7A). In contrast, for the familiarity judgment task, the results of the permutation test showed that the semantic information was represented in both VWFA-1 and VWFA-2 (Figure 7B). These results reveal that the posterior VWFA subregion represents the visual information, while the anterior VWFA subregion represents the semantic information during Chinese character reading. In addition, the processing demands modulate the representation of lexical information of VWFAs. In particular, the posterior VWFA (VWFA-1) undergoes a functional switch from lexical to semantic processing under higher semantic processing demands.

Taking advantage of the Chinese characters and RSA, Experiment 2 explored the neural representation of lexical information (visual, phonological, and semantic information) in two VWFAs and the modulatory effects of processing demands. Consistent with previous studies in alphabetic language (White et al., 2023; Zhan et al., 2023), results showed that the posterior VWFA was sensitive to low-level visual information, while the anterior VWFA was responsible for the representation of high-level semantic information. In addition, processing demands modulate the representational patterns of VWFAs. These results provided evidence for the local functional differences across VWFA subregions from the perspective of functional modularization.

## DISCUSSION

Using multimodal neuroimaging techniques (resting-state fMRI, task-state fMRI, and dMRI), the present study investigated functional properties and connectivity patterns of VWFA subregions (i.e., VWFA-1 and VWFA-2). Activation analysis revealed that Chinese characters elicited extensive activation of dorsal and ventral reading networks. The results of both structural and functional connectivity consistently indicated that the posterior VWFA (VWFA-1) showed a stronger connection with the orthographic network, while the anterior VWFA (VWFA-2) showed a stronger connection with the semantic network. In line with the connectivity patterns, the results of representational similarity analysis showed that the VWFA-1 was sensitive to low-level visual information, while the VWFA-2 was sensitive to high-level semantic information during Chinese word reading. These convergent findings elucidated the neural activity of local brain regions in VWFA and their cooperative mechanism with distant brain regions related to language processing, jointly providing direct neuroimaging evidence for the connectivity-biased hypothesis.

The present study contributed three significant findings on the function of VWFA subregions during word reading. First, by adopting classical localizer tasks to accurately define the orthographic, phonological, and semantic networks, the present study revealed the functional and structural connectivity patterns between VWFA subregions and reading-related networks. Although previous studies have demonstrated distinctive connectivity patterns between VWFA subregions and language networks, they mainly used a unimodal imaging approach, providing limited insight into the neural mechanism (Chen et al., 2019; Li et al., 2020). In comparison, using classical localizer tasks combined with multimodal neuroimaging techniques (i.e., resting-state fMRI and dMRI), the present study explored the functional and structural connectivity patterns between VWFA subregions and the language networks. Specifically, the seed-based whole-brain functional connectivity results indicate that the VWFA-1 showed more robust functional connectivity with the orthographic-related network, while the VWFA-2 showed more robust functional connectivity with the phonological and semantic-related network. Consistently, ROI-to-ROI analysis results further confirmed similar functional connection preferences between VWFA subregions and the language networks. More importantly, using probabilistic fiber tracking analysis, we found similar results that the VWFA-1 showed more robust structural connectivity with the orthographic network, while the VWFA-2 showed more robust structural connectivity with the phonological and semantic network. These converging results are in line with previous findings on structural (Bouhali et al., 2014; Yeatman & White, 2021) and functional connectivity (Li et al., 2020; Yablonski et al., 2024), jointly providing multimodal neuroimaging evidence of the functional differentiation in the anterior and posterior VWFA subregions from the perspective of connectivity.

It is worth mentioning that the rsFC between the semantic network and the VWFAs was negative. The VWFA was reported to be primarily involved in the visual-orthographic processing (Chauhan et al., 2024; Van Audenhaege et al., 2025), whereas the semantic network (i.e., SFG, IFG, middle temporal gyrus, and posterior temporoparietal cortex in the present study) is responsible for conceptual meaning representation (Arrigo et al., 2024; Bucur & Papagno, 2021; He et al., 2025). The negative rsFC between the semantic network and the VWFAs may reflect functional segregation between two regions or a dynamic balance between the visual word form processing system and the semantic system in their spontaneous activity. Although the rsFC is negative, the rsFC to the semantic network from VWFA-2 was significantly stronger than that of VWFA-1 (*p* < 0.001), indicating that there is a functional differentiation between the anterior and posterior VWFA subregions.

The second contribution of the present study is to disentangle the neural representation of lexical information in VWFA subregions and clarify the modulatory effect of processing demands. VWFA has been reported as a highly heterogeneous structure, and its subregions show different engagement in orthographic, phonological, and semantic processing (Caffarra et al., 2021; Zhan et al., 2023). However, the specific information representation in VWFA subregions remains unclear because the use of alphabetic language has relatively obvious GPC rules (Fischer-Baum et al., 2017; Lo & Yeh, 2018). Therefore, it is difficult to clearly disentangle the contributions of orthography and phonology to neural activations in lvOT (Glezer et al., 2016; Gu et al., 2024). Although several studies used Chinese characters that deviated from the GPC rules as materials (Liu et al., 2023; Zhao et al., 2017), the limited quantity of such materials resulted in a low ecological validity. To address these issues, the present study used a relatively large number of Chinese characters as materials, combined with RSA to explore the lexical information representation in VWFA subregions. Consistent with previous research (Cohen et al., 2021; Zhan et al., 2023), the present study found that the posterior VWFA (VWFA-1) represented the visual information of Chinese characters, while the anterior VWFA (VWFA-2) represented the semantic information of Chinese characters during the structure judgment task. These results characterized the functional divisions of VWFA subregions during Chinese reading. In addition, we found that when performing the familiarity judgment task with higher processing demands of semantic information, both the anterior and posterior VWFA subregions represent the semantic information of Chinese characters. These results were consistent with previous findings (Branzi et al., 2022; Li et al., 2023), indicating that processing demands modulate the representation of lexical information in VWFA. In particular, under higher semantic processing demands, the posterior VWFA (VWFA-1) undergoes a functional switch from lexical to semantic processing, which is attributed to top-down modulation, which reflects the dynamics of brain function (Qin et al., 2021; Schlaffke et al., 2015; Song et al., 2012; Wang et al., 2018).

Notably, the representation of phonological information was found in neither the structural judgment nor the familiarity judgment tasks. It might be attributed to two reasons. First, previous studies have reported that Chinese adopts a logographic script and relies less on phonological information (Li et al., 2022; Wong et al., 2014). Second, the structural judgment and familiarity judgment tasks have been reported to focus more on visual and semantic information, respectively (Gu et al., 2024; Li et al., 2023). Therefore, the low processing demands for phonological information result in a weaker representation of phonological information. Future research could utilize tasks with higher phonological processing demands (e.g., naming task) to further explore the neural mechanisms of phonological processing.

The third contribution of the present study is to provide multimodal neuroimaging evidence for the connectivity-biased hypothesis. According to the connectivity-biased hypothesis, the function of brain regions is associated with their connectivity network patterns (Dehaene & Dehaene-Lambertz, 2016; Humphreys et al., 2021). Several studies have explicitly investigated functional connectivity (Li et al., 2020), structural connectivity (Chen et al., 2019), and the functional role (Gu et al., 2024) of VWFAs. However, the associations between connectivity patterns and the functional role of VWFAs have not been comprehensively explored. The present study adopted multimodal neuroimaging techniques (i.e., task-state fMRI, resting-state fMRI, and diffusion MRI) combined with RSA to investigate the functional role of VWFA subregions and the brain circuitry supporting their function. Results showed respective roles of the posterior and anterior VWFA subregions in visual and semantic processing, which is consistent with their respective connectivity to orthographic and semantic networks. These convergent findings elucidated the local neural computations in the VWFAs and their cooperative mechanism with distant brain regions related to language processing, jointly providing multimodal neuroimaging evidence for the connectivity-biased hypothesis (Humphreys et al., 2021). These findings also suggested that clarifying the function of brain regions in word reading requires a comprehensive consideration of their functional characteristics and connectivity patterns.

Two limitations should be addressed in future studies. First, we only conducted the localizer task on a subset of participants and mainly defined the language networks and two VWFAs at the group level, which may obscure individual differences. Future research could conduct the localizer task for all participants and define the language networks and VWFAs at the individual level to obtain more precise and meaningful results. Second, although the present study found consistency between the function of VWFA and its connectivity patterns, the causal relationship between its function and connectivity patterns remains to be determined. Transcranial magnetic stimulation (TMS) is a well-established tool for establishing the causal role of specific brain regions in cognitive processes (Bergmann et al., 2021; Trajkovic et al., 2025), and has been used to reveal the functional role of VWFA in word reading (Pattamadilok et al., 2019). Therefore, future research could further investigate the causality between connectivity patterns and function by modulating the activity of the language network or VWFA through methods such as TMS.

## CONCLUSION

The present study elucidated the functional specialization of the posterior and anterior VWFA subregions in visual and semantic processing, and the brain circuitries supporting their function in word reading through multimodal neuroimaging integration and RSA. These convergent findings of the local neural computations in the VWFA subregions and their cooperative mechanism with distant brain regions related to language processing provide neuroimaging evidence for the connectivity-biased hypothesis.

## Funding Information

This study was supported by grants from the Guangdong Basic and Applied Basic Research Foundation (2024A1515011023), the National Natural Science Foundation of China (32271098, 32571226), Research Center for Brain Cognition and Human Development, Guangdong, China (no. 2024B0303390003), and Striving for the First-Class, Improving Weak Links and Highlighting Features (SIH) Key Discipline for Psychology in South China Normal University.

## Author Contributions

**L.G.**: Data curation (Equal); Formal analysis (Lead); Methodology (Equal); Visualization (Equal); Writing – original draft (Equal); Writing – review & editing (Lead). **H.L.**: Conceptualization (Equal); Data curation (Equal); Methodology (Equal); Visualization (Equal); Writing – original draft (Equal); Writing – review & editing (Lead). **J.Q.**: Conceptualization (Equal); Data curation (Equal); Methodology (Equal); (Supporting); Visualization (Supporting); Writing – original draft (Supporting); Writing – review & editing (Supporting). **J.Y.**: Formal analysis (Equal); Visualization (Supporting); Writing – original draft (Supporting); Writing – review & editing (Supporting). **X.F**.: Writing – review & editing (Supporting). **R.H.**: Writing – review & editing (Supporting). **L.M.**: Conceptualization (Lead); Methodology (Lead); Visualization (Lead); Writing – original draft (Lead); Writing – review & editing (Lead).

## Code and Data Availability Statements

The materials, statistical code, and data are available at OSF (https://osf.io/n9kqj/?view_only=8768b3b808b94d149d9843615df35b35).

## Supplementary Material



## TECHNICAL TERMS

lvOT: The left ventral occipitotemporal cortex.
fMRI: Functional magnetic resonance imaging.
RSA: Representational similarity analysis.
rsFC: Resting-state functional connectivity.
