# Supplementary material for: Functional Specialization of the Visual Word Form Area During Word Reading: A Multimodal Neuroimaging Study
Source: Neurobiol Lang (Camb). 2026 Mar 26;7:NOL.a.225. doi: 10.1162/NOL.a.225 (PMC13065096; doi:10.1162/NOL.a.225)
Supplement: Supplementary file 1 [file nol-07-225-s001.pdf]

Supplementary Materials for Functional specialization of the visual word form area during word reading: a multimodal neuroimaging study

Contents

A The definition of language networks in Exp 1 **2**

B Details on individual-level VWFA in Exp 1 **3**

C Brain activation results in Exp 2 **10**

D The visual representation results of the occipital cortex in Exp 2 **11**

E The representation results of the lexical information in Exp 2 **12**

A The definition of language networks in Exp 1

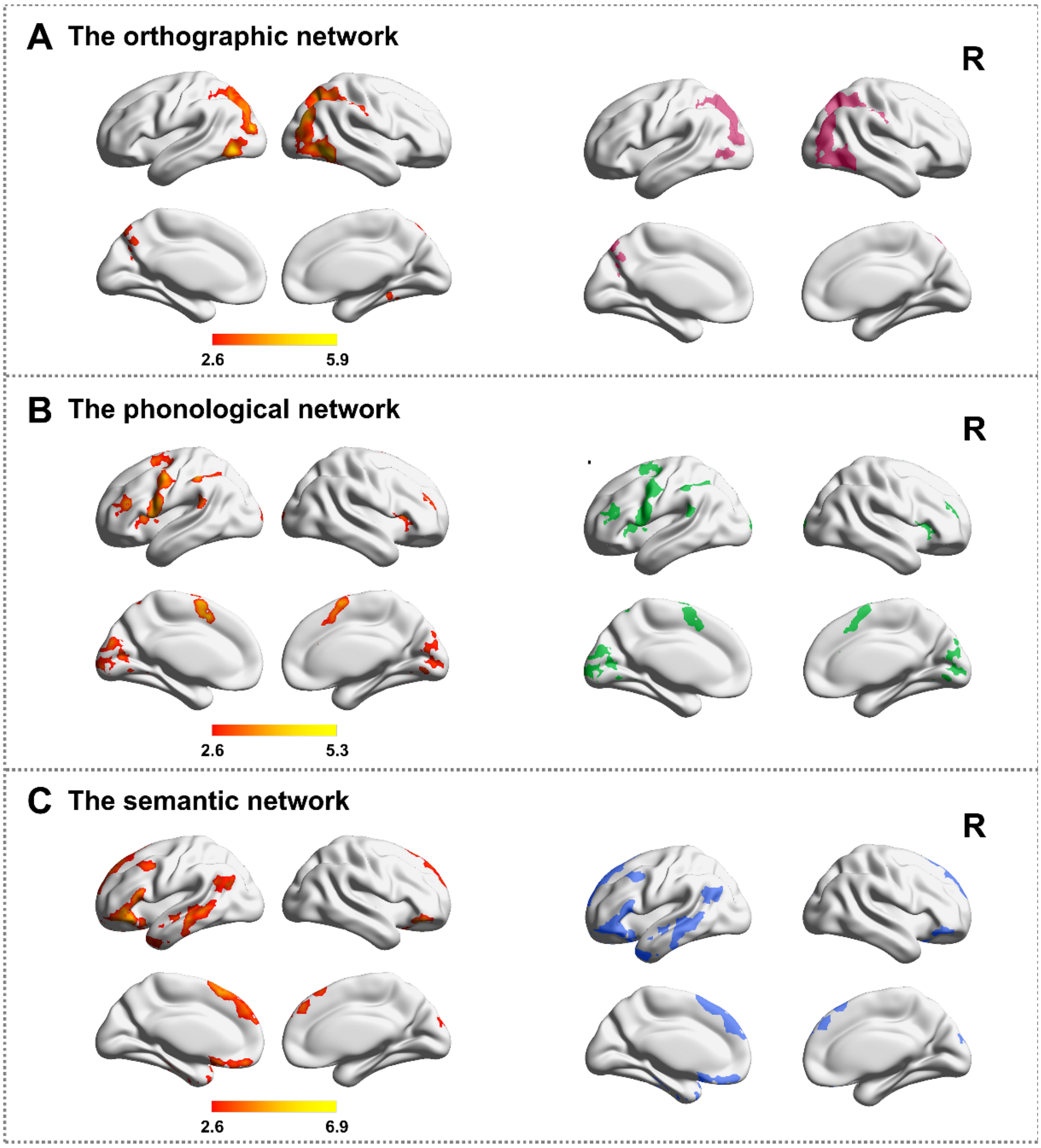

Supplement Fig. 1 The orthographic (A), phonological (B), and semantic (C) networks defined based on the localizer task. The orthographic network was defined by the contrast of radical > rhyme + semantic judgment task (pinkish red), the phonological network was defined by the contrast of rhyme > radical + semantic judgment task (green), and the semantic network was defined by the contrast of semantic > rhyme + radical judgment task (blue). R = right.

B Details on individual-level VWFA in Exp 1

During the VWFA localizer task, participants viewed sequences of stimuli from 4 different categories: Chinese characters, scrambled images, false fonts, and facial images (Supplement Fig. 3). Each type of material had 52 items. Chinese characters and scrambled images were used to localize the VWFAs. The Chinese characters consisted of 5–13 strokes (mean = 8.33 ± 0.31) with high usage frequency (mean = 581.52 ± 60.86 per million words) (Cai and Brysbaert, 2010). Scrambled images were created by scrambling the Chinese character images with a 4-pixel tile size (Fischer-Baum et al., 2017).

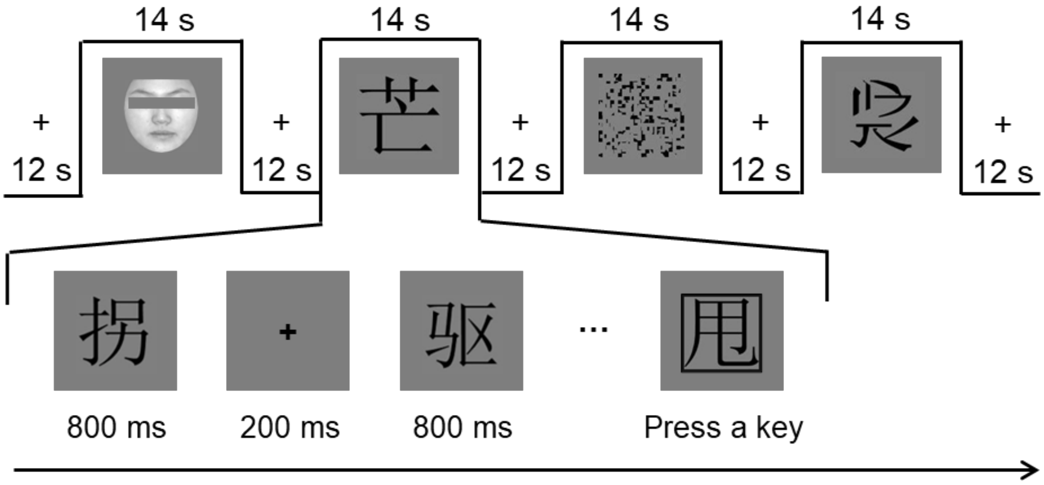

Supplement Fig. 2 Experimental materials and design for the VWFA localizer task. During the localizer scan, participants were instructed to press a key whenever they noticed the word with a black frame.

The VWFA localizer task was a block design with 2 runs. Each run consisted of 16 blocks, with 4 blocks for each category of materials. Blocks were arranged in Latin

square order. Task blocks were interspersed with 12s fixation blocks. There were 14 trials in each block. In each trial, the stimulus was presented for 0.8s, followed by a 0.2s fixation (Supplement Fig. 3). To ensure that participants were attentive to the stimuli, they were instructed to press a button when they saw a stimulus that was framed with black lines. Each participant completed two runs of the VWFA localizer task, which lasted for 15 minutes.

Activation analysis of the VWFA localizer task was consistent with that used for the orthographic, phonological, and semantic networks localizer tasks. Specifically, the contrast of Chinese characters minus scrambled images was computed for each participant and for each run, then a fixed-effects model was used to get the integrated imaging data across two runs for each participant. Subsequently, we functionally defined individuals' VWFAs based on the greater activation for Chinese characters relative to scrambled images at the individual level. Specifically, we raised the threshold from $Z = 1.96$ (corresponding to a one-tailed test with $p < 0.025$) until the number of contiguous surviving voxels reached 50-100 around the coordinates of VWFA-1 and VWFA-2 reported in White et al. (2023). Two participants failed to define the VWFA and were excluded from subsequent analysis. For the remaining 75 participants, the mean number of voxels was 98 in the VWFA-1 and 97 in the VWFA-2 (Supplement Table 1). The mean MNI coordinates were -42.5, -61.8, 12.0 for VWFA-1 and -43.2, -45.7, -18.2 for VWFA-2, which were both close to the classical coordinates.

Supplement Table 1. **The information of VWFA-1 and VWFA-2 defined at the individual level.**

Cluster Size (Voxels)	Z	MNI coordinates			Cluster Size (Voxels)	Z	MNI coordinates		
VWFA-1		x	y	z	VWFA-2		x	y	z
100	2.59	-42	-62	-11	100	2.60	-44	-47	-18
100	3.31	-42	-62	-10	100	3.11	-46	-46	-19
100	1.99	-43	-60	-12	100	2.20	-46	-47	-17
55	1.97	-43	-69	-17	98	1.99	-32	-49	-19
100	2.01	-42	-61	-20	95	1.96	-40	-32	-21
100	3.60	-42	-62	-10	100	2.54	-45	-47	-19
88	1.97	-45	-62	-10	69	1.99	-40	-44	-19
100	2.35	-42	-62	-11	100	2.56	-42	-45	-17
100	2.13	-44	-61	-11	100	2.09	-41	-46	-17
100	2.83	-43	-65	-10	100	2.31	-45	-48	-21
100	1.97	-41	-60	-13	100	2.50	-45	-47	-18
100	2.17	-40	-62	-13	100	2.10	-43	-47	-18
100	2.22	-42	-63	-8	83	1.99	-44	-47	-15
100	2.27	-41	-60	-12	59	1.96	-43	-49	-17
100	2.75	-44	-61	-11	100	2.24	-42	-49	-16
87	1.96	-43	-59	-11	100	2.12	-43	-47	-19
100	2.16	-43	-62	-10	100	2.49	-44	-47	-16
100	2.30	-42	-63	-11	100	2.13	-42	-48	-17
100	1.99	-48	-61	-19	100	2.35	-45	-45	-19
100	2.21	-42	-62	-10	100	2.41	-43	-48	-17
100	2.36	-44	-63	-14	100	1.96	-43	-50	-16
100	2.04	-42	-62	-11	100	2.00	-45	-44	-20
100	2.36	-46	-66	-10	100	2.15	-43	-45	-22
100	3.67	-42	-62	-10	100	2.36	-44	-44	-17

Cluster Size (Voxels)	Z	MNI coordinates			Cluster Size (Voxels)	Z	MNI coordinates		
VWFA-1		x	y	z	VWFA-2		x	y	z
100	2.20	-42	-63	-10	100	2.56	-46	-46	-19
100	2.44	-42	-63	-15	100	1.98	-44	-46	-20
100	1.98	-45	-63	-10	100	2.07	-44	-47	-19
100	2.03	-41	-62	-9	100	2.01	-44	-50	-19
100	1.98	-43	-61	-11	100	2.41	-44	-48	-18
100	1.96	-43	-62	-13	100	2.61	-43	-45	-18
84	1.96	-48	-61	-9	98	1.99	-44	-48	-18
100	2.33	-43	-60	-14	100	3.33	-46	-48	-19
100	4.22	-42	-62	-10	100	2.41	-46	-48	-18
100	3.17	-42	-62	-11	100	2.00	-34	-45	-19
100	2.08	-44	-63	-14	100	2.10	-44	-43	-21
100	2.24	-42	-62	-11	100	2.10	-43	-48	-18
98	1.98	-42	-62	-12	100	1.97	-45	-48	-18
100	1.97	-46	-61	-13	100	2.12	-44	-47	-18
100	3.02	-42	-62	-10	91	1.97	-46	-50	-18
100	2.67	-46	-50	-18	98	1.98	-43	-45	-19
100	2.00	-42	-61	-10	99	1.97	-43	-48	-19
100	2.21	-43	-63	-9	99	2.50	-45	-51	-16
100	2.21	-43	-62	-11	100	2.66	-45	-48	-18
100	2.37	-42	-62	-10	100	2.14	-45	-47	-18
100	2.39	-44	-61	-11	98	2.01	-44	-49	-17
100	2.16	-44	-67	-9	100	2.19	-43	-48	-23
100	2.14	-41	-64	-13	64	1.97	-39	-39	-19
100	2.26	-43	-62	-14	100	1.96	-43	-49	-20
100	2.09	-42	-62	-9	100	2.36	-46	-46	-18
100	2.45	-42	-61	-11	100	2.21	-44	-48	-18
100	2.90	-38	-76	-12	36	1.96	-39	-48	-22

Cluster Size (Voxels)	Z	MNI coordinates			Cluster Size (Voxels)	Z	MNI coordinates		
VWFA-1		x	y	z	VWFA-2		x	y	z
100	2.14	-43	-61	-10	100	2.13	-41	-51	-12
100	2.45	-42	-62	-11	100	2.51	-43	-45	-22
100	1.96	-44	-61	-20	100	2.07	-39	-41	-21
100	2.06	-44	-59	-14	100	2.11	-42	-49	-16
100	2.14	-43	-62	-10	100	2.68	-45	-46	-18
100	2.04	-44	-61	-12	100	2.07	-44	-47	-17
92	1.98	-42	-52	-11	74	2.02	-40	-46	-20
100	2.00	-44	-62	-14	100	2.07	-44	-46	-18
100	2.21	-43	-64	-10	100	3.41	-44	-47	-18
97	1.98	-42	-53	-21	93	1.96	-48	-33	-20
73	1.97	-42	-65	-15	100	2.32	-45	-45	-19
100	2.14	-40	-74	-13	100	2.73	-41	-46	-13
50	1.96	-41	-53	-18	100	2.71	-40	-39	-21
100	2.15	-38	-56	-17	100	2.33	-39	-34	-20
100	2.41	-42	-62	-10	100	2.52	-45	-47	-17
100	2.78	-42	-62	-9	100	2.08	-43	-48	-15
100	2.84	-43	-60	-11	100	2.13	-46	-46	-18
100	3.58	-42	-61	-10	100	2.58	-46	-48	-17
100	1.99	-43	-62	-12	99	1.96	-44	-46	-18
100	2.46	-42	-63	-12	100	2.37	-45	-44	-16
100	2.11	-42	-62	-9	100	2.17	-45	-46	-18
100	2.07	-31	-62	-16	100	2.97	-38	-45	-19
100	1.98	-42	-62	-11	100	2.15	-44	-45	-18
100	3.46	-43	-62	-10	99	1.96	-46	-47	-19

Then, we conducted the identical analytical approach used for the population-based VWFA to examine both the functional and structural connectivity analysis between the

individual-based VWFAs and the orthographic, phonological, and semantic networks.

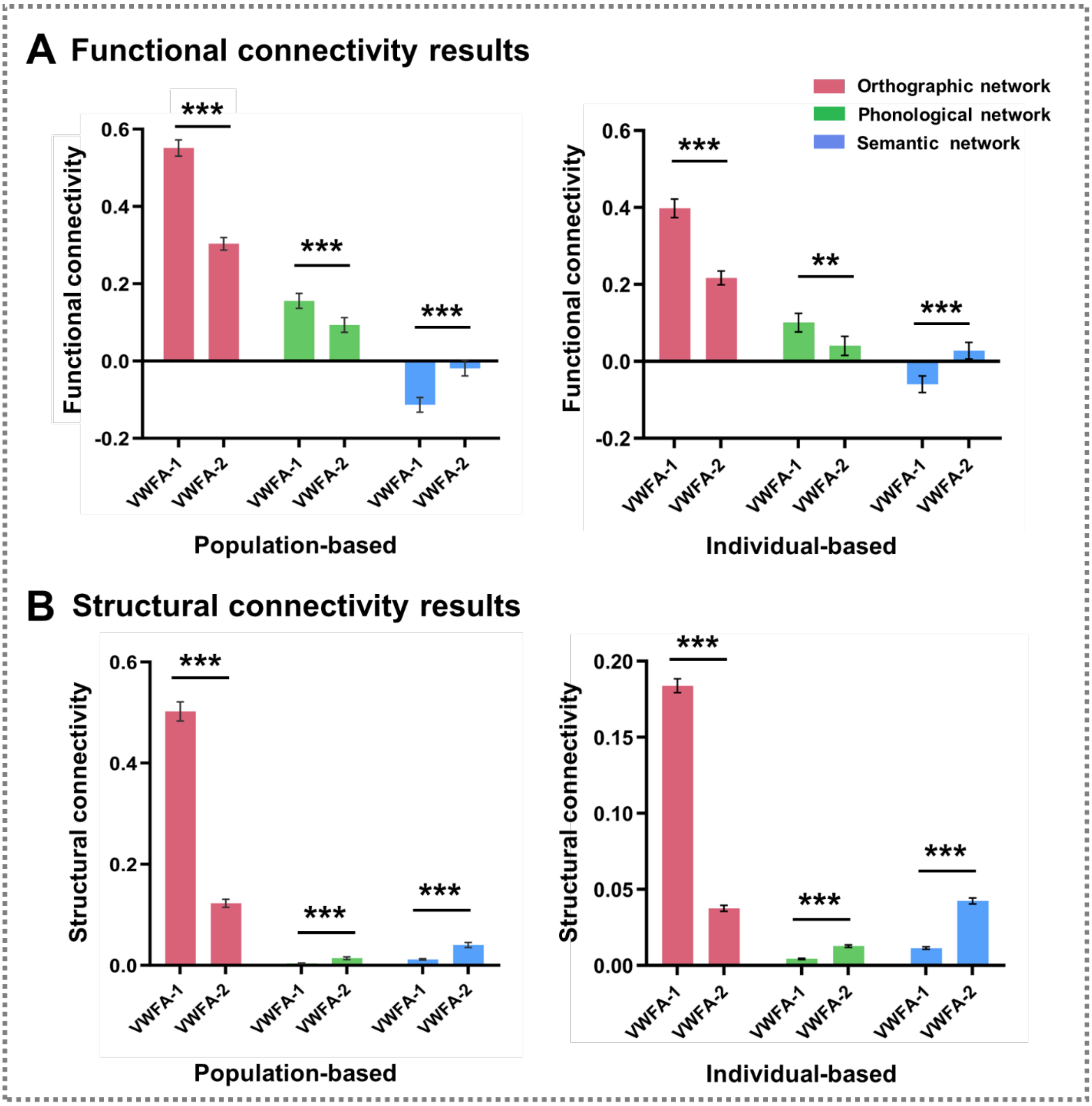

Supplement Fig. 3 The resting-state functional connectivity (A) and the structural connectivity (B) results between the language networks and the two VWFAs defined at the population level (the left panel) and the individual level (the right panel). The histograms show the strength of the connectivity between the VWFAs and orthographic (pinkish red), phonological (green), and semantic (blue) networks. Error

bars represent the standard error of the mean. ***: $p < 0.001$. **: $p < 0.01$.

Results showed that the patterns of both functional and structural connectivity between the individual-based VWFAs and the language networks were consistent with those observed for the population-based VWFA (Supplement Fig. 4). These results indicate that, at least in the context of the present study, the structural and functional connectivity between the VWFA (based on both the individual-level and population average MNI coordinates) and the language network are relatively robust.

References:

Cai Q, Brysbaert M. SUBTLEX-CH: Chinese Word and Character Frequencies Based on Film Subtitles. Plos One 2010, 5: e10729.

Fischer-Baum S, Bruggemann D, Gallego IF, Li DSP, Tamez ER. Decoding levels of representation in reading: A representational similarity approach. Cortex 2017, 90: 88-102.

White AL, Kay KN, Tang KA, Yeatman JD. Engaging in word recognition elicits modulations in visual cortex. Current Biology 2023, 33: 1308.

C Brain activation results in Exp 2

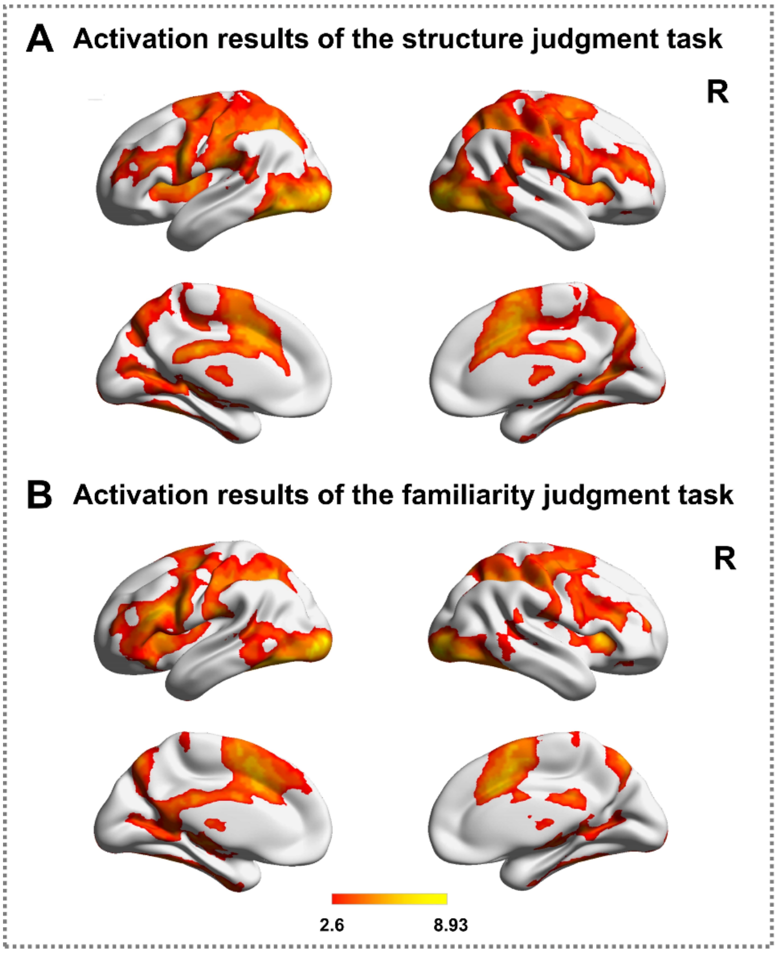

Supplement Fig. 4 Brain activations for Chinese during the structure judgment task (A) and the familiarity judgment task (B). R = right.

D The visual representation results of the occipital cortex in Exp 2

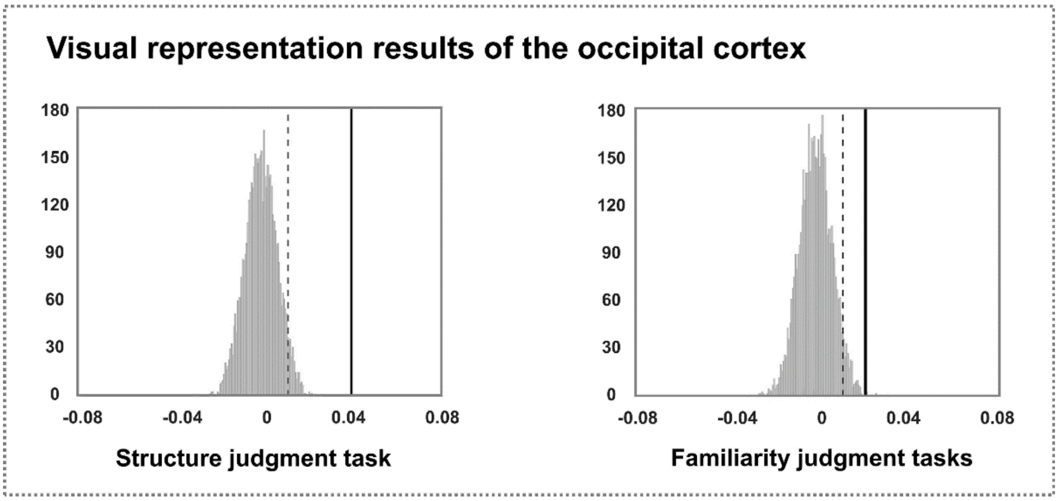

Supplement Fig. 5 The visual representation results of the occipital cortex in the structure judgment (the left panel) and the familiarity judgment (the right panel) task. Spearman correlations were calculated between the neural DSMs and the visual DSM. The correlation coefficients were Fisher Z-transformed. A 5000-permutation test was performed and a 95% confidence interval was used. The x-axis represents the z value after Fisher's Z transform. The solid black line indicates the actual z value of the representational similarity, and the dotted black line indicates the fifth percentile (0.05, one-tailed) of the distribution.

E The representation results of the lexical information in Exp 2

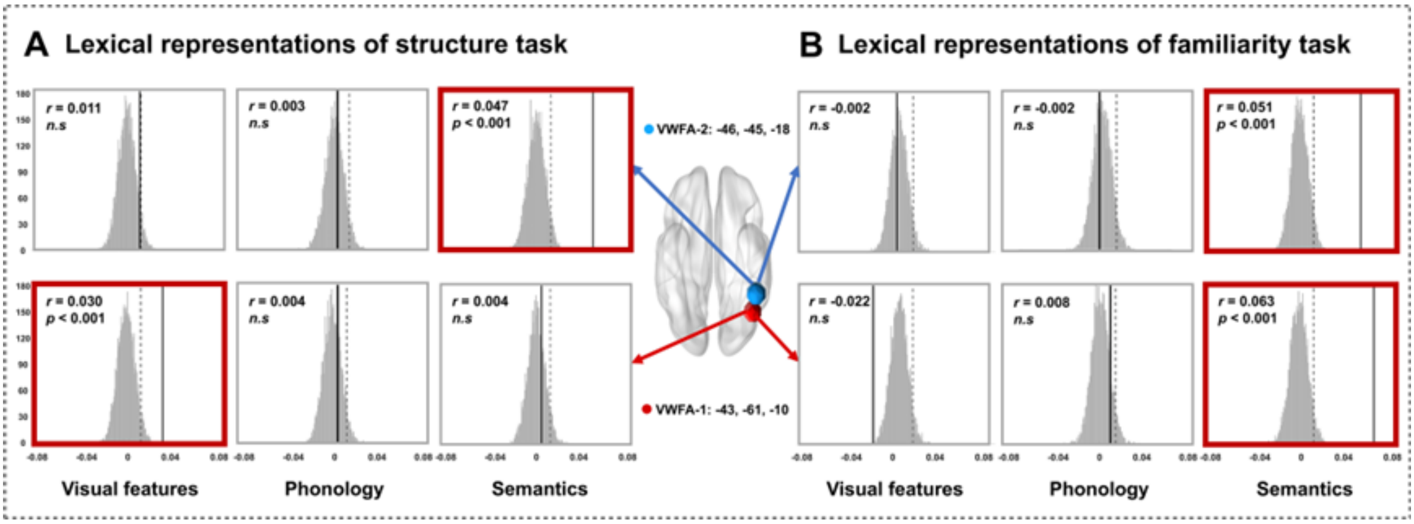

Supplement Fig. 6 The representation results of the lexical information in the structure judgment task (A) and the familiarity judgment task (B). The MNI coordinates of VWFA-1 were -43, -61, -10, and those of VWFA-2 were −46, −45, −18. Spearman correlations in two ROIs were calculated between the neural DSMs and the visual, phonological, and semantic DSMs, respectively. The correlation coefficients were Fisher Z-transformed. A 5000-permutation test was performed and a 95% confidence interval was used. The x-axis represents the z value after Fisher's Z transform. The solid black line indicates the actual z value of the representational similarity, and the dotted black line indicates the fifth percentile (0.05, one-tailed) of the distribution.
